# Dual functionality nanobioconjugates targeting intracellular bacteria in cancer cells with enhanced antimicrobial activity

**DOI:** 10.1038/s41598-017-06014-4

**Published:** 2017-07-19

**Authors:** Rohini Singh, Smita Patil, Neetu Singh, Shalini Gupta

**Affiliations:** 1 Department of Chemical Engineering, Indian Institute of Technology Delhi, Hauz Khas, 110016 India; 2 Centre for Biomedical Engineering, Indian Institute of Technology Delhi, Hauz Khas, 110016 India

## Abstract

Bacterial drug resistance has emerged as a serious global threat mandating the development of novel methodologies that allow facile modulation of antimicrobial action in a controlled fashion. Conjugating antibiotics to nanoparticles helps to meet this goal by increasing the drug’s overall avidity, bioavailability and easier internalisation into mammalian cells, targeting bacteria that otherwise escape antibacterial action by host cell-localisation. We used polymyxin B sulfate (PMB) and sushi peptide as model drugs against Gram-negative bacteria and established their enhanced antimicrobial activity on *Escherichia coli* (*E. coli)* cells after conjugation to gold nanoparticles (AuNPs). The efficacy of the bioconjugates was also tested on *Salmonella typhi (S. typhi)* bacteria infected into cervical cancer cells (HeLa) and further improved through specific targeting via folate receptors. Our results demonstrate significantly lower inhibitory concentration values for sushi-NP assemblies as compared to free drug, especially at optimal drug loading levels. No major cytotoxicity was observed in mammalian cells alone.

## Introduction

Antibiotics are the most conventional therapeutics used for the treatment of bacterial infections, however, 50% of these drugs prescribed to humans are either not needed or not effectively utilized as prescribed. Consequently, misuse of antibiotics is the most important factor leading to the catastrophic threat of antibiotic resistance around the world^1^. Conventional antibiotics suffer from several issues such as improper biodistribution, poor water solubility, lack of target specificity and loss of efficacy over time due to the emergence of drug resistance in pathogenic bacteria^2^. To overcome these issues, higher doses of antimicrobials are often prescribed, which further worsen the situation as the bacteria evading the action of drug become even more resistant over time. The process is more critical in Gram-negative bacteria as their cell wall structure is quite complex containing a thick lipid layer, which when degraded, has the potential to cause tremendous pathogenicity^3^. Since no new major antibiotics have been developed in the last 40 years, except for the recent discovery of synthetic antibacterial agents oxadiazoles^4^, a new strategy is perhaps required for improving the efficacy of conventional antibiotics and dealing with the resistance crisis. The efficacy can either be improved by developing a more potent derivative of a drug molecule or by improving its delivery and interaction within the bacteria. The use of a delivery vehicle can dramatically lower the antibiotic minimum inhibitory concentration (MIC) or half maximal inhibitory concentration (IC_50_) value by presenting the drug molecules in such a way that it facilitates the interaction of the active groups with the target molecules on the bacteria, increasing its overall efficacy^5–8^. Typically, nanoparticles (NPs) are used for improving the delivery and specificity of therapeutics^2, 9, 10^.

Antibiotic resistance poses an even bigger problem in diseases like cancer where patients are at a higher risk of developing serious bacterial infections due to prolonged neutropenia, lymphocyte dysfunction, mucositis, and use of invasive devices^10–13^. Additionally, since chemotherapy cannot specifically target bacteria, if the bacterial infection in cancer remains untreated, the bacteria can infect the host even after the cancer cells are killed by chemotherapy. Thus, it becomes even more crucial to ensure elimination of live bacteria from the tumor region. Substantial use of antibiotics here again builds selection pressures, which ultimately leads to the emergence of resistant microorganisms^14^. In the immunologically impaired cancer patients, the first dose of antibiotics administered is extremely important. A recent study showed that combination antibacterial and chemotherapy treatment lead to notable reduction of tumour activity and marked survival benefit over either therapy alone^15^. Also, bacteriophages (viruses that infect and lyse bacteria), matched for specific bacterial isolates, have been used in the past to treat antibiotic-resistant infections in cancer patients but the therapy does not offer a facile option in acute settings due to challenges with affordability, limited host range, phage-resistance, side effects from bacterial lysis and several other regulatory issues^16–18^. Thus, new combative strategies are desperately required to deal with bacterial infections in cancer patients.

Recently, peptides have emerged as a new class of antimicrobials with lesser cytotoxic effects than conventional antibiotics^19^. They are recognized for being highly selective and efficacious and, at the same time, relatively safe and well-tolerated. Consequently, commercial pharmaceutical research is fast shifting its focus toward this new class of antibiotics and close to 140 peptide therapeutics are currently under clinical trials^20^. Despite being an attractive alternative to conventional antibiotics, the usage of peptides also has limitations in stability and delivery, which needs to be addressed before their full potential is realized. As most peptide therapeutics are injectables, there exist challenges relating to their acidic and enzymatic degradation and inefficient internalization into cellular membranes^20^. In addition, peptides are prone to hydrolysis and oxidation, and have a higher tendency to aggregate and undergo faster elimination. Use of NP delivery platforms can overcome this rapid degradation, instability and aggregation issues, crossing of biological membrane barrier and increasing retention time^5^.

**Figure 1:**
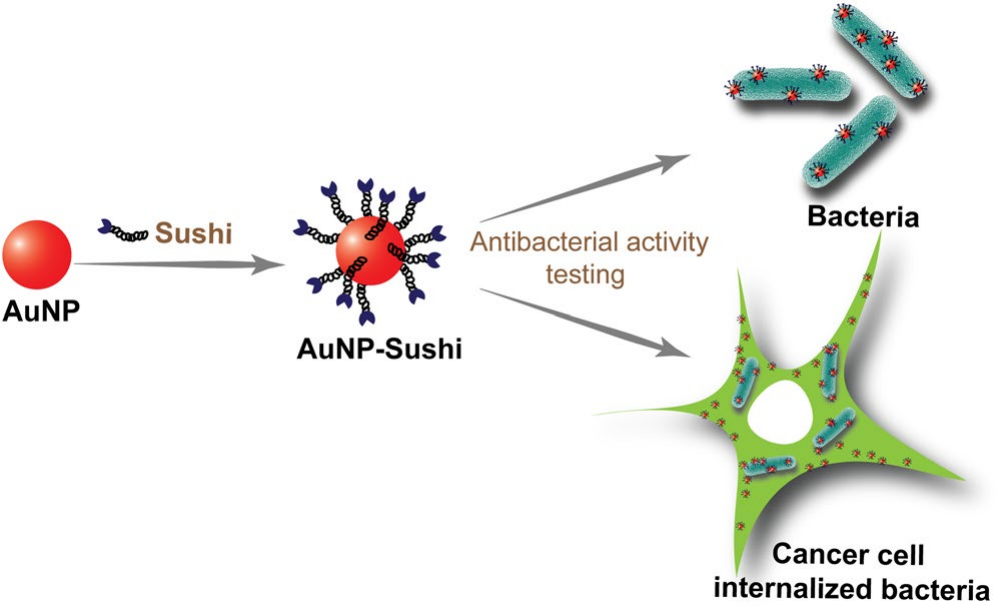
Schematic of approach: Sushi peptide molecules in the free and NP-conjugated forms tested for their antimicrobial activity on extracellular and HeLa-internalized bacterial cells.

In this paper, we demonstrate a versatile strategy using AuNPs as carrier platforms to improve the antibacterial efficiency of a potent drug/peptide. In order to target the more common Gram-negative bacterial infections, we have worked with a relatively less known but potentially valuable antibiotic called sushi peptide and compared its response to a more widely known but highly neuro- and nephrotoxic antibiotic called polymyxin B sulfate (PMB). The structure-property relationship studies for these drugs illustrate that while PMB acts by neutralizing and shedding off the cell wall component lipopolysaccharide (LPS) present on the outer cell wall of all Gram-negative bacteria^21^, sushi uses LPS as a latch and lyses the bacteria by disrupting the membrane and releasing its cytosolic content, without LPS leakage^22^. This may be beneficial as free LPS in the bloodstream is known to cause medical complications such as sepsis. The response of the two drugs were tested and compared in the free and NP-conjugated forms (Fig. 1). The system was further applied to intracellularized bacteria inside cervical cancer cells (HeLa cells) by taking advantage of the folate receptors present on these cancer cells. In humans, folate receptors are overexpressed in breast, brain, liver, lung and ovarian carcinomas, whereas the receptor is expressed in low levels in thyroid and kidney as well as present in choroid plexus and placenta^23, 24^. Here we have used HeLa cells as a model system as they overexpress folic acid receptors^25, 26^. Since, folate receptors are upregulated in various human cancer cell types^27^ the internalization of folate conjugated NPs can be extended to many drugs for lowering the antimicrobial dosages. The extent of drug loading was found to be a crucial factor in modulating the particle’s antibacterial activity.

## Results

**Figure 2:**
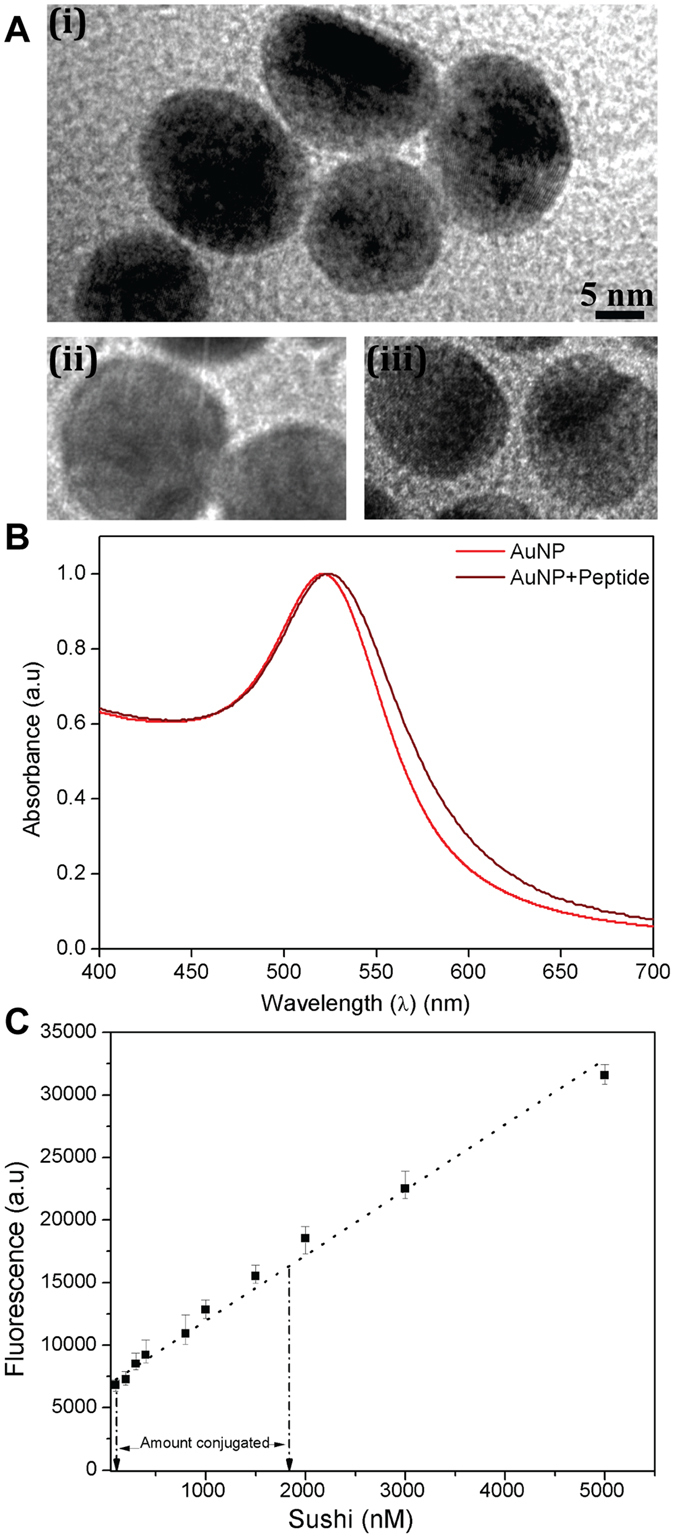
(**A**) TEM images of unmodified AuNPs (i) and AuNPs after conjugation with peptide (ii) and PMB (iii). (**B**) UV-visible spectra of AuNPs depicting a red-shift in their peak absorbance after conjugation with sushi peptide (Δλ_max_ = 4 nm). (**C**) OPA calibration charts for quantifying the amount of peptide molecules linked to the AuNPs. (Linear least square fits).

AuNPs were synthesized, and their morphology, size and stability was characterized by different techniques including transmission electron microscopy (TEM) (Fig. 2A) and UV-Visible spectroscopy (Figs 2B, S3A), which showed that the particles were fairly spherical, monodisperse (16 ± 4 nm) and stable for several months when stored at 4 °C. The NP surface modification with PMB/sushi lead to a red-shift in the extinction peak suggesting successful ligand binding. The binding of PMB, sushi and folic acid to AuNPs was also qualitatively confirmed by FTIR (Fig. S1). Further, the stoichiometric molar ratio between the appended antimicrobial molecules sushi/PMB and NPs was estimated using a fluorescent derivatization reagent, ortho-pthaldehyde (OPA), that enabled fluorescence detection and quantification of primary amines^22, 23^ present in the system before and after conjugation (Figs 2C and S3B)^28, 29^. Using this approach, the binding efficiencies for different starting molar ratios of PMB/sushi and NPs were estimated as summarized in Table 1. To rule out the possibility that the reduction in the fluorescence signal is not an artefact of the sushi peptides settling by themselves during the centrifugation process, we performed an experiment by centrifuging the free peptides and then measuring the fluorescence of the supernatant and comparing with the sushi-OPA calibration curve (Fig. S2). The results implied that the free peptides did not settle in solution and so, the amount of conjugated peptide was reasonably established in our experiments.

**Table 1:** Binding efficiencies of PMB and sushi peptides on AuNPs.

AuNP-PMB		AuNP-sushi	
Relative molar ratio (NP:PMB)	Conjugation efficiency (%)	Relative molar ratio (NP:sushi)	Conjugation efficiency (%)
1:10	∼100%	1:500	∼98%
1:20	∼100%	1:700	∼98%
>1:20	Aggregation	1:1000	∼95%
		1:2000	∼90%
		>1:2000	Aggregation

**Figure 3:**
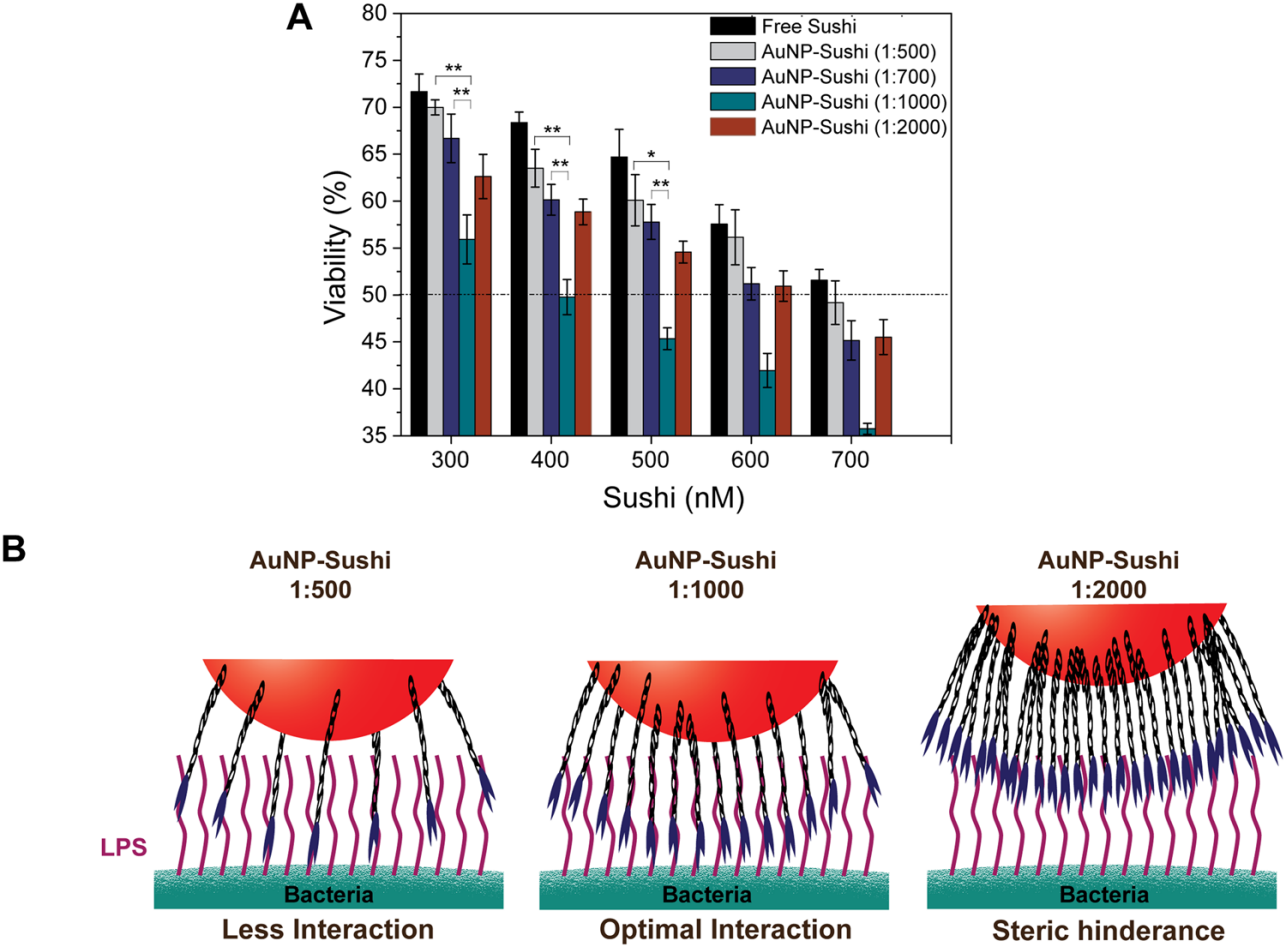
Antibacterial activity analysis of AuNP conjugates with *S. typhi* (**A**) NP-sushi conjugates are superior to free drug at all concentrations especially at 1:1000 molar ratio that gives ∼42% reduction in IC_50_ value. (Two way ANOVA, **P < 0.01, *P < 0.05). (**C**) Proposed model for NP-sushi and bacterial LPS interaction.

The different conjugates were applied to our model *E. coli* and *S. typhi* bacteria in a microtitre broth assay at 37 °C and pH 7.4. Free drug and particles alone (without drug) were used as controls. After overnight incubation, the changes in absorbance due to condition-dependent bacterial growth were measured. The results suggest that PMB when delivered with the AuNPs killed ∼10% more *E. coli* in comparison to free PMB (Fig. S4A). In comparison, the results with sushi on *S. typhi* and *E. coli* showed a considerably more dramatic trend where the efficacy of the NP-bioconjugates first increased and then decreased non-monotonically as the relative molar ratio of the peptides was increased on the AuNPs (Figs 3A and S4B). Conjugates with 1000x molar excess displayed the most favourable performance lowering the IC_50_ values by as much as 42% (∼400 nM) when compared to the free peptide (∼700 nM) and reached MIC at ∼700 nM. Interestingly, the conjugates with 1000x molar excess showed significantly higher antibacterial activity in comparison with conjugates having lower molar excess of peptides.

**Figure 4:**
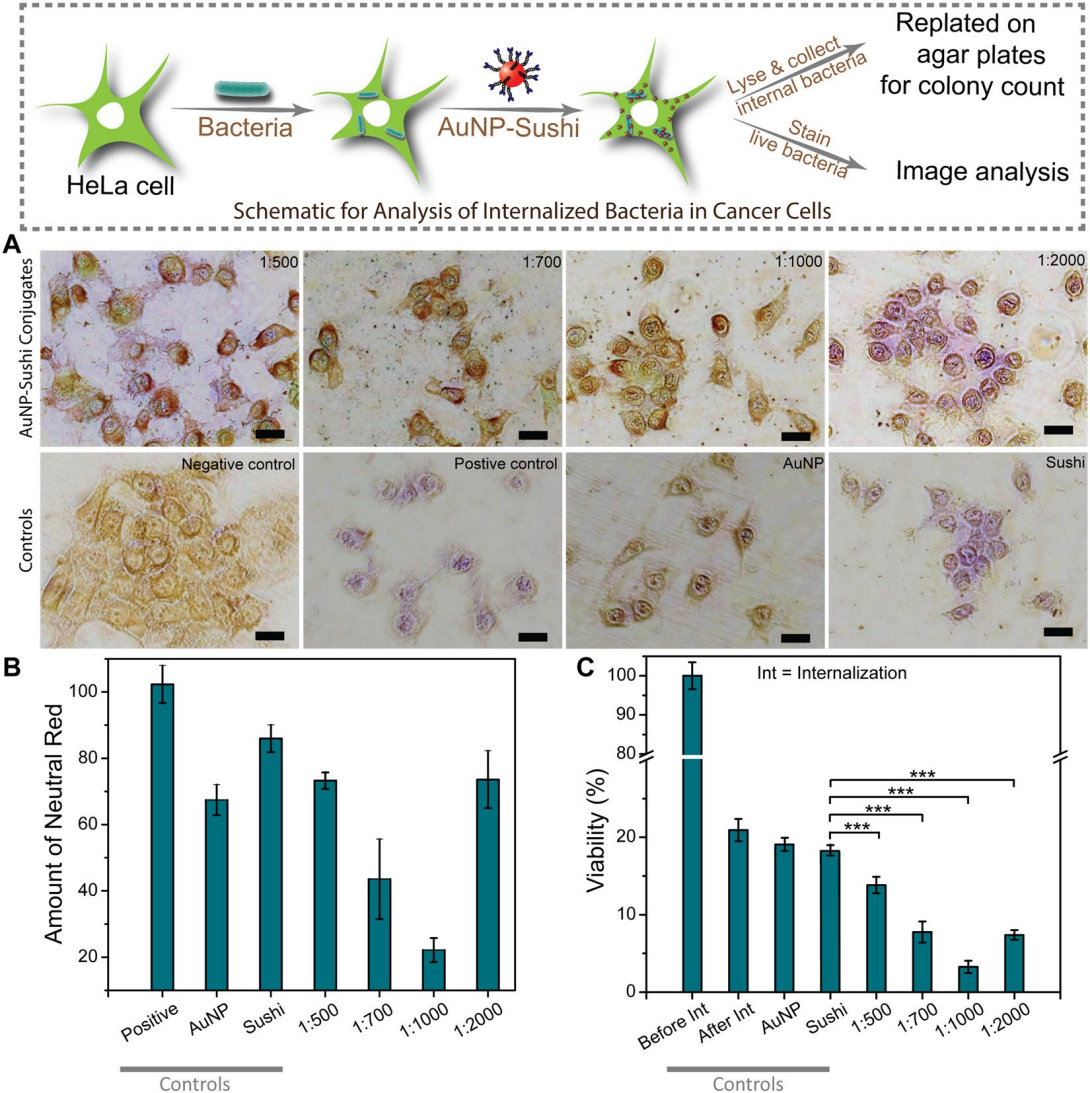
HeLa cell infection assays with *S. typhi* internalization. (**A**) Optical microscopy images: Load-dependent NP-sushi conjugate activity confirming maximum antibiotic effect at 1:1000 ratio. The total peptide concentration was fixed at 4 µM in all the cases. Purple color indicates live bacteria at the time of neutral red dye incubation. Negative control: HeLa cells alone, Positive control: HeLa cells with only *S. typhi*, AuNP: HeLa cells with only AuNPs, Sushi: HeLa cells with only sushi peptide. Scale bar: 20 μm. (**B**) Quantification of neutral red using ImageJ software, (**C**) Percent viability by colony count of *S. typhi* bacteria internalised in HeLa cells after antibacterial treatment with free and conjugated sushi peptides.

The conjugated scaffolds were tested for their cytotoxicity behaviour in mammalian cells through MTT assay and were found to be well tolerated (Fig. S5). The results of the assays confirmed that the AuNP-sushi conjugates at different molar ratios up to 2 μM total peptide concentration were non-cytotoxic to the HeLa cells and were functionally very similar to that of free drug. The NP conjugates were then tested against the cell-internalizing bacteria *S. typhi*. The internalized live *S. typhi* bacteria were analysed by optical microscopy after staining with neutral red dye and fixing of the infected HeLa cells. The results showed that the least number of bacteria survived in the 1:1000 case as compared to other ratios (Fig. 4A and B) corroborating with our previous results. Control experiments performed with no drug, free sushi peptide at the same concentration (4 µM) and AuNPs without any drug showed purple-stained live bacteria as anticipated. To compare these results quantitatively, we lysed the mammalian cells and plated the lysate on LB agar plates. Colony counts obtained by plating were again in accordance with the observations of the live bacteria staining analysis. All the AuNP-sushi conjugates showed significantly higher antibacterial activity on internalized bacteria as compared to free sushi peptide at the same concentration (Fig. 4C), which is in agreement with our antibacterial study on free bacteria. Once again, the lowest viability was found in 1:1000 AuNP-sushi conjugates. Analysis of cell survival by calcein AM staining of HeLa cells after *S. typhi* infection followed by 5 h exposure to peptides and free/conjugated AuNPs revealed that HeLa cells were viable even after the bacterial infection (Fig. S6), showing the conjugates had no major toxic behaviour against mammalian cells. Along with this, a real time monitoring of bacterial cell death study was also performed in case of sushi peptides on *E. coli* cells to obtain the time frame in which the bacterial death starts (Fig. S7) and the results showed that the significant killing of bacteria was observed within less than one hour leading to complete death in 5 hours.

**Figure 5:**
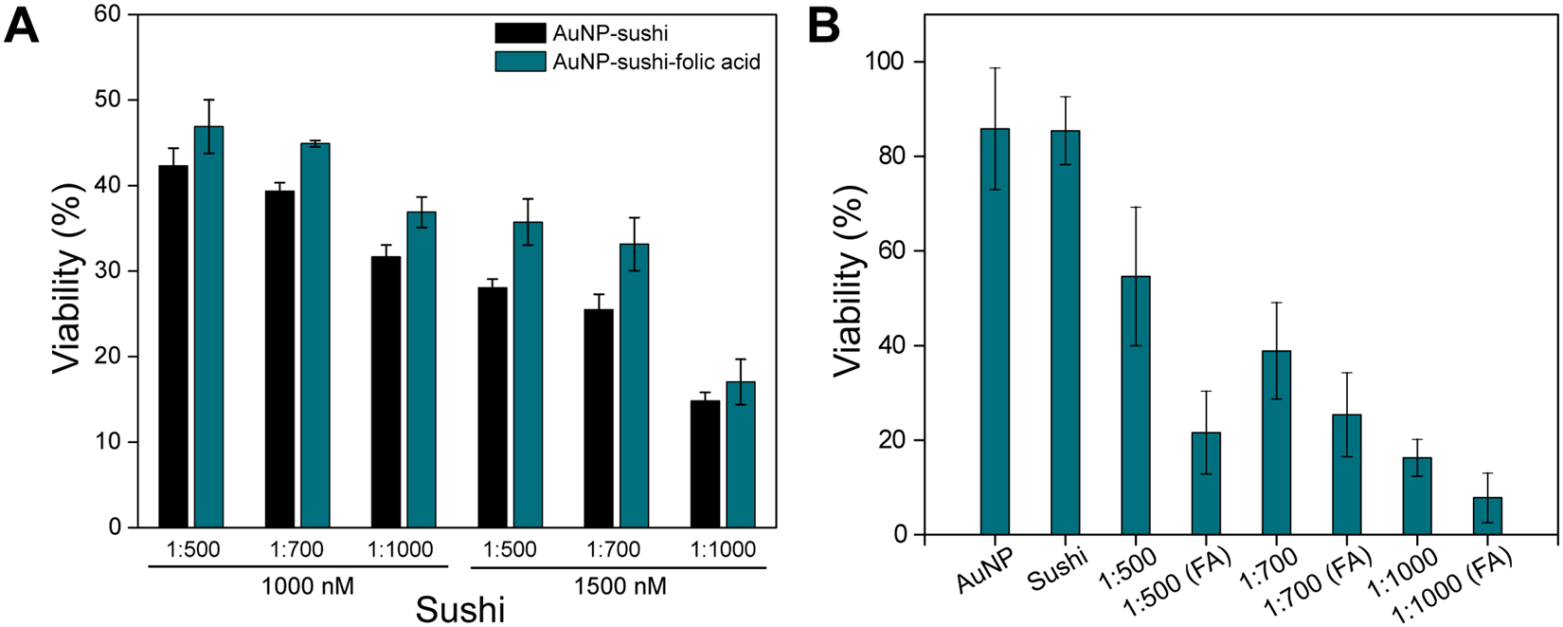
Comparison of antimicrobial activity against *S. typhi* for AuNP-sushi with and without FA-functionalization. Percent viability by colony count of bacteria before (**A**) and after (**B**) internalization inside HeLa cells. (One way ANOVA, ***P < 0.0001).

The AuNP-sushi were conjugated to the folic acid ﻿(FA) molecules via the amine groups present on the peptides. This conjugation was confirmed by UV-spectroscopy as FA gives a characteristic peak at 280 nm (Fig. S8). The effect of FA conjugation on the antibacterial activity of AuNP-sushi conjugates was then studied against *S. typhi* before and after internalization into HeLa cells. The results revealed that in comparison to AuNP-sushi, FA-conjugated NPs had a slightly lower antimicrobial efficacy for non-internalized *S. typhi* irrespective of the total drug used or the ratio of the peptides taken per particle (Fig. 5A). This is expected since the FA molecules are attached to the NPs on top of the already anchored sushi peptides. On the other hand, the viability of the HeLa-internalized bacteria lowered considerably after exposure to the FA-conjugated NPs, especially for the 1:500 ratio NPs, proving our hypothesis correct (Figs 5B and S9). The amount of AuNPs internalised in the mammalian cells was quantified using the inductively coupled plasma mass spectrometry (ICP-MS) technique (Fig. S10) which clearly showed higher uptake of FA conjugated NPs by HeLa cells.

## Discussion

We began our studies by evaluating the antimicrobial susceptibility of conjugates on bacterial cells before and after their conjugation to gold NPs (AuNPs). Gold was chosen due to its low reported toxicity, ease of synthesis and functionalization with small molecules^30, 31^. Quantification of the extent of drug binding allowed us to determine the optimum drug loading required for achieving the highest antibacterial activity amongst all the NP-bioconjugates. Sushi peptides are biologically-active derivatives of the widely popular factor C protein found in horseshoe crabs^32^. Factor C proteins are extensively used in endotoxin detection assays such as the popular endotoxin test such as LAL tests^33, 34^. As a result, the sushi peptides have a well-established and specific interaction with the LPS, although its role in clinical applications is still not fully explored. There are two reported forms of sushi peptides, S1 and S3, both of which are 34-mer with a strong affinity for LPS^35^. While the S1 type forms a random coil structure and functions as a monomer transforming into alpha helix, S3 generally exists in the dimeric form and forms alpha and beta sheets on interaction with LPS^36^. In the literature, it is reported that the binding efficiency of S3 increases as it goes from the monomeric to dimeric to tetrameric forms, i.e., a locally high concentration of peptides has a synergistic effect on its binding properties^37^. Therefore, we selected the S3 type sushi for our study. The site for NP conjugation was purposely designed to be terminally opposite to the LPS-binding side in order to cause minimal interference in the peptide’s activity. We hypothesized that when the peptides attach to the NPs, they behave as multimeric units providing a much larger and suitable surface area for interaction with the LPS on the bacterial membrane. It is possible that some of the peptides also leach out from the AuNP surface but for the large part, we believe that it is the peptides that remain associated to the AuNPs surface that disintegrate the cell membrane ultimately causing bacterial death. Au-thiol linkage is known to be quite stable above pH 7 with a bond strength (50 to 100 kJ/mol) close to that of a weak covalent bond^38, 39^.

The suitability of interaction depends both on the intermolecular spacing between the peptides and their relative position with respect to the LPS molecules. If the peptides are spaced too far apart from each other as shown in Fig. 3B, the binding may be sub-optimal. Similarly, at very high packing densities, surface crowding may lead to steric effects such as incomplete presentation of the lysine (present at the 5^th^ and 7^th^ positions in the peptide sequence from the N-terminal side) and thus, show relatively less efficient performance. Based on geometrical considerations, we roughly estimated the peptide-peptide and peptide-LPS intermolecular distances in the intermediate case where the peptides were in 1000x molar access to NPs. Our results indicated that indeed, more peptides are available for LPS-binding in this case as the outer edges of the peptides lie at distances that are commensurate with the intermolecular LPS gap sizes (see Fig. S11 and Table S1 for detailed calculations). These calculations assumed LPS molecules to be aligned parallel to each other as is often the case in a real scenario; LPS molecules form tightly packed islands on the outer cell wall of the bacteria and constitute around 3 to 4 million copies per cell. In summary, the 1:1000 NP-sushi conjugates were the most favourable for antimicrobial susceptibility studies amongst all the bioassemblies. The NP-sushi also becomes a better choice than NP-PMB as they offer a wider window for function-modulation. Further, unlike PMB, sushi lyses the bacteria by disrupting the cell wall but does not allow the bacteria to shed its pathogenic components into the bloodstream which can be a huge advantage in real systems^22^.

### Antimicrobial testing on intracellularized bacteria

Intracellular bacteria are a great threat to human well-being as they often go undetected and avoid elimination by the macrophages^40^. Many replicate within the phagocytic vacuoles while others escape to enter the cell cytosol where the free drugs have a lower retention time and get flushed out even before they have a chance to interact with the bacteria^41^. After establishing the improved efficacy of the sushi NP conjugates for antimicrobial activity, we proceeded to our next objective, i.e., to see how efficiently these particles may be used for cancer cell targeting of internalized bacteria. This required evaluating the particles’ cytotoxic behaviour and ability to retain antibiotic activity inside mammalian cells.

Cancer cells provide a facile opportunity to improve cellular internalization by exploiting the surface receptors overexpressed on them. We utilized folic acid (FA) conjugation on the AuNP-sushi conjugates to enhance their internalization and further improve their antibacterial activity. The overexpressed folate receptors (FR) on cancer cells increases the internalization of NPs containing FA via receptor mediated endocytosis^42–44^. Many studies have shown that modifying NPs with FA ligands improves targeting and uptake of drugs by cancer cells which has a huge potential for selective delivery of chemotherapeutic agents to FR-positive cancer cells^27, 44–46^. In our case, the sushi-AuNPs were conjugated to the FA molecules via the amine groups present on the peptides. Results from folic acid experiments suggest that at all the concentrations studied, FA conjugated NPs were more effective against intracellular bacterial infection in cancer as it enhanced the internalization of NPs in cancer cells via folate receptors. This enhanced antibacterial activity due to better internalization of AuNP-sushi conjugates can allow administration of lower doses of antimicrobials for Gram-negative infections in cancer and thus help in combating antimicrobial resistance. This should also aid in patient survival as the bacteria would not get released into the host upon killing of cancer cells during chemotherapy. In future, our strategy could effectively be used for a combinatorial antibacterial and chemotherapy treatment by conjugating a chemotherapeutic to the AuNP-sushi-folic acid NPs, which will kill cancer cells as well as the intracellular bacteria, thus avoiding further spread of bacterial infection to normal cells.

We have demonstrated an efficient antibiotic targeting strategy using AuNPs that increases both the antibacterial efficacy and particle uptake for destroying intracellular Gram-negative bacterial infection. Sushi peptides are more potential antimicrobials providing the window for modulation of antibacterial activity without affecting the mammalian cells even at relatively higher concentrations. In addition, the FA conjugated NPs proved to be efficient in targeting the cancer cells infected with pathogenic bacteria and showed increased antibacterial activity as compared to non-folic acid conjugated NPs. We anticipate that this strategy can be applied in future for preparation of NPs based target specific multivalent scaffolds which might help in lowering the dosage as well reducing the chances of drug resistance development. Also conjugating NPs with ligands specific for a particular type of cell/organ in which infection is prominent, especially in organ specific cancer, can be studied further and this might help in development of more potent and target specific surface engineered drug delivery vehicles.

## Methods

### Reagents

Bacterial strains of *E. coli* (DH5α) and *S. typhi* (serovar *enterica*) were obtained from the School of Biological Sciences at IIT Delhi. The S3 sushi peptide sequence (HAEHKVKIGVEQKYGQFPQGTEVTYTCSGNYFLMC, M.W. 4,083 Da, purity > 95%) was synthesized by Genemed Synthesis (San Antonio, USA). Gold(III) chloride trihydrate (HAuCl_4_.3H_2_O), sodium citrate dihydrate (C_6_H_5_Na_3_O_7_.2H_2_O), tannic acid (C_76_H_62_O_48_), polymyxin B sulfate (PMB), 4-(2-hydroxyethyl)-1-piperazineethanesulfonic acid (HEPES), ortho-pthaldehyde (OPA), Luria broth (LB), folic acid (FA), 1-ethyl-3-(3-dimethylaminopropyl) carbodiimide (EDC), N-hydroxysuccinimide (NHS) and boric acid were purchased from Sigma Aldrich (India). Dithiolalkane aromatic PEG6 hydrazide (DTH) was obtained from Sensopath Technologies (USA). Neutral red and gluteraldehyde (GLA) were obtained from CDH (India). Dulbecco’s modified eagle medium (DMEM, high glucose), fetal bovine serum (FBS), 3-(4,5-Dimethylthiazol-2-yl)-2,5-diphenyltetrazolium bromide (MTT), Dulbecco’s phosphate-buffered saline (PBS), antibiotic-antimycotic solution and calcein AM were purchased from Invitrogen (India). All solutions were prepared using ultrapure deionised (DI) Milli-Q water (∼18.8 mΩ.cm resistivity) (CDUFBI001, Millipore, USA). HeLa cell line was obtained from the National Centre for Cell Science (NCCS) (Pune, India).

### Synthesis of AuNPs conjugates

AuNPs of approximately 16 nm size were synthesized using the Turkevich method^47, 48^. For this, 5 mL of 1% w/v HAuCl_4_ was mixed with 395 mL of DI water. Simultaneously, a reducing solution was prepared by mixing 20 mL of 1% w/v sodium citrate, 50 µL of 1% w/v tannic acid and 80 mL of DI water. The gold and the reducing solution were then heated up to 60 °C separately and mixed together under constant stirring at 60 °C for 4 h. Once the solution turned wine red color indicating the formation of AuNPs, the suspension was quenched in an ice-bath and stored at 4 °C. Next, the AuNPs were functionalized with sushi peptides or PMB drug. The AuNP-sushi conjugates were prepared in a 10 mL batch by mixing the peptides with 2 nM AuNP suspension in different molar ratios. The mixtures were incubated overnight at 25 °C inside a shaker incubator (Sciengenics orbitek) at 100 rpm. The conjugated particles were purified by three consecutive centrifugal washes at 8690, 11175 and 14900 rcf for 20 min each and the pellet was resuspended in 40 mM HEPES buffer at pH 7.4. All the particle conjugates were stored at 4 °C until further use and were stable for at least a month.

The AuNP-PMB conjugates were prepared using a protocol reported earlier^49^. DTH was added to 10 mL of AuNP suspension in a molar ratio of 1:10^4^ (AuNP:DTH) and incubated overnight (Sciengenics orbitek) at 25 °C under constant shaking at 100 rpm. The DTH-functionalized AuNPs were purified via centrifugation at 8690, 11175 and 14900 rcf for 20 min each and finally resuspended in 40 mM HEPES buffer at pH 7.4 containing 1% v/v GLA. This mixture was kept overnight at 25 °C inside the shaker incubator at 150 rpm. The GLA-DTH-AuNP suspension was again washed three times as above and resuspended in the HEPES buffer. Finally, PMB was added in different molar ratios and the mixture was incubated overnight at 25 °C at 150 rpm. In the end, PMB-conjugated AuNPs were retrieved in 40 mM HEPES buffer at pH 7.4 by following the same washing steps as stated above. The particle conjugates were stable at 4 °C for at least 6 weeks.

### Particle characterization

The size of the AuNPs was determined using the Tecnai G2 transmission electron microscope (TEM). For this, the 4x AuNP suspension was dried on a carbon-coated copper TEM grid (CF 200 CU, Electron Microscopy Sciences, USA). The samples were imaged at 200 kV and the micrographs obtained at 44000X were analyzed using the open source ImageJ software. The average size of the NPs from the particle size distribution curves was obtained to be 16 ± 4 nm. The extent of peptide/PMB conjugation on the AuNPs was measured using the OPA fluorescence detection technique. For this, 8 mg of OPA reagent was added to 100 μL of 95% v/v ethanol. This OPA solution was mixed with 200 µL of β-mercaptoethanol and 10 mL of 0.4 M boric acid titrated with KOH to pH 9.7. Equal volumes of the drug/peptide and OPA solutions were mixed and the fluorescence emission at 455 nm was measured at an excitation peak of 340 nm using a microplate reader (BioTek Synergy H1). A calibration chart was prepared by taking different concentrations of drug/peptide and the extent of sushi/PMB binding was estimated by measuring the fluorescence signal response in the supernatant before and after conjugation. For attenuated total reflection-fourier-transform infrared (ATR-FTIR) spectroscopy, the spectra of the liquid sample (50 µL) were directly recorded for sushi and AuNPs conjugated with either sushi or both sushi and folic acid.

### Antimicrobial activity assays

The antimicrobial tests were performed by the microdilution broth assay in 96-well cell culture plates (Nest). *E. coli* cells were grown overnight in LB broth (3 mL broth in 15 mL tube) at 37 °C and 250 rpm. The concentration of the bacteria was determined by counting colony forming units (CFU) on LB agar plates as well as absorbance at 625 nm in UV-VIS spectrophotometer (Shimadzu UV-2600). The cells were diluted to 2 × 10^6^ CFU/mL and 50 μL of this suspension was mixed with 100 μL of two times concentrated LB and 50 μL of free PMB/sushi or conjugated NPs in different molar concentrations. The samples were incubated overnight at 37 °C and the bacterial growth was measured the next day by taking absorbance readings at 625 nm (BioTek Synergy H1 microplate reader). All the samples were prepared in triplicate and each experiment was repeated three times. The final data were reported as a mean of all the values ± 1 SD (standard deviation). Two way ANOVA was performed for calculating the statistical significance (** for p < 0.01, * for p < 0.05). The bacterial growth without any antibiotic was taken as positive control and all the test results were normalized with it to calculate the viability.

### Mammalian cell culture

The HeLa cells were cultured in high-glucose DMEM with 10% FBS (called complete media from here on). When 90% confluent, the cells were detached by adding trypsin and then centrifuged and re-dispersed in complete media. The suspended cells were seeded in a 96-well cell culture plate (Nest) at a density of 10,000 cells/well. For imaging purpose, cells were also cultured on glass coverslips kept inside 24-well cell culture plates (Nest) at a seeding density of 50,000 cells/well. The culture plates were kept overnight in a CO_2_ incubator at 37 °C with 5% CO_2_.

### MTT assays

MTT assays were carried out to assess the cytotoxicity of free and conjugated drug/peptides. After incubating the cells for 24 h with different concentrations (in complete medium) of the free PMB/sushi or NP conjugates, cells were washed with PBS and then 20 μL of 5 mg/mL MTT in PBS was added to the 200 μL of complete medium in each well. The plate was incubated for 1 h to allow the formation of purple formazan crystals inside the cells. The media were replaced by DMSO to dissolve the crystals and their concentration was determined by taking optical density (O.D.) measurements at 550 nm (BioTek Synergy H1). All the data were presented as mean value ±1 standard deviation from at least three independent experiments performed in triplicate. Cells incubated with only complete medium were used as positive controls and other values were normalized using positive control.

### HeLa cell infection assays

*S. typhi* bacteria were grown overnight in LB broth at 37 °C and 250 rpm. The late logarithmic cultures were diluted in complete medium without serum and used to infect the HeLa cell monolayer using a multiplicity of infection of 200 bacteria per cell. The bacterial cells were left to internalize by the cells for 2 h at 37 °C after which the medium was replaced with 2x concentrated antibiotic-antimycotic solution to kill any remaining extracellular bacteria. After washing with PBS, the HeLa cells were incubated with free or conjugated sushi peptides for 3.5 h at 37 °C in order to study their effect on the intracellularized bacteria. AuNPs alone were used as negative control. The plated cells were washed three times with PBS and then lysed using 100 μL of 0.1% w/v SDS per well (in the 96-well plate format). The number of viable bacteria was determined by plating 10 μL of the lysed sample onto LB agar plates and counting the growth after overnight incubation at 37 °C. For image analysis, the mammalian cells were incubated with 30 μL of neutral red stain in 300 μL of complete medium at 37 °C for 20 min and then fixed using 4% w/v paraformaldehyde in PBS for 20 min. The cells were then imaged in bright field mode using an Olympus IX 73 microscope fitted with a Q-imaging digital color camera.

### Cell viability assays

The viability of the infected HeLa cells was analyzed by a live/dead staining assay. The assays were carried out by staining the cultured cells with 0.3 μL of 2 μM calcein AM per 300 μL of complete medium at 37 °C. After 30 min of incubation, the stained samples were washed thrice with PBS and visualized using fluorescence microscopy with FITC filter (Olympus IX 73). The appearance of a bright green color indicated viable cells whereas; the dead cells didn’t show any fluorescence under the microscope. Images were taken using a high speed digital camera (Orca Flash 4.0, Hammamatsu) after fixing the exposure time.

### Synthesis of FA conjugates and their antimicrobial activity

1 mg/mL stock of FA was prepared in 50 mM NaOH. A final solution of 1000 µM FA concentration, pH- 7.4, was prepared in 1 mg/mL each of EDC and NHS in HEPES buffer and was kept for 1.5 h incubation at room temperature at 100 rpm. The FA solution with activated carboxylic groups was then added to AuNP-sushi conjugated suspension in 100 molar excess (AuNP:FA) to all the synthesized ratios (AuNP:sushi) and was kept for overnight incubation at room temperature and 100 rpm. The conjugated particles were purified by three consecutive centrifugal washes at 8690, 11175 and 14900 rcf for 20 min each and the pellet was resuspended in 40 mM HEPES buffer at pH 7.4. Absorbance spectra of the synthesized FA-conjugated NPs was studied using UV-VIS spectrophotometer (Shimadzu UV-2600). Antibacterial activity of FA-conjugated and non-conjugated AuNP:sushi of different ratios on *S. typhi* bacteria was studied using microdilution broth assay in 96-well cell culture plates. The procedure was similar to the one as described in antimicrobial activity assays method section. The final concentrations of all the NPs used was 1000 and 1500 nm. The effect of folic acid conjugated NPs on intracellular bacteria was studied by colony count method as described previously. The final concentration of all the particles as well sushi peptides used for the study was 4000 nm. The data were normalized against a positive control - HeLa cells infected with internalized bacteria.

### Inductively coupled plasma mass spectrometry (ICP-MS)

100 µL solution of AuNPs or conjugated NPs prepared in complete media was added onto each well of 96 well plate containing overnight grown HeLa cells (10000 cells/well). Samples were incubated at 37 °C for 4 h and then washed three times with PBS, trypsinized and the cells were counted using a haemocytometer. Finally, the cells were centrifuged and digested in 100 µL aqua regia and analysed by Agilent’s 7900 ICP-MS instrument.

## Electronic supplementary material

Supplementary information
