# Supplementary material for: Dual functionality nanobioconjugates targeting intracellular bacteria in cancer cells with enhanced antimicrobial activity
Source: Sci Rep. 2017 Jul 19;7:5792. doi: 10.1038/s41598-017-06014-4 (PMC5517437; doi:10.1038/s41598-017-06014-4)
Supplement: Supplementary file 1 — Supplementary information [file 41598_2017_6014_MOESM1_ESM.pdf]

# Dual functionality nanobioconjugates targeting intracellular bacteria in cancer cells with enhanced antimicrobial activity

*Rohini Singh*[δ,#], *Smita Patil*[ξ,#], *Neetu Singh*[ξ,*], *Shalini Gupta*[δ,*]

[δ]Dept. of Chemical Engineering, Indian Institute of Technology Delhi, 110016, India
[ξ]Centre for Biomedical Engineering, Indian Institute of Technology Delhi, 110016, India

## ***Supplementary Information***

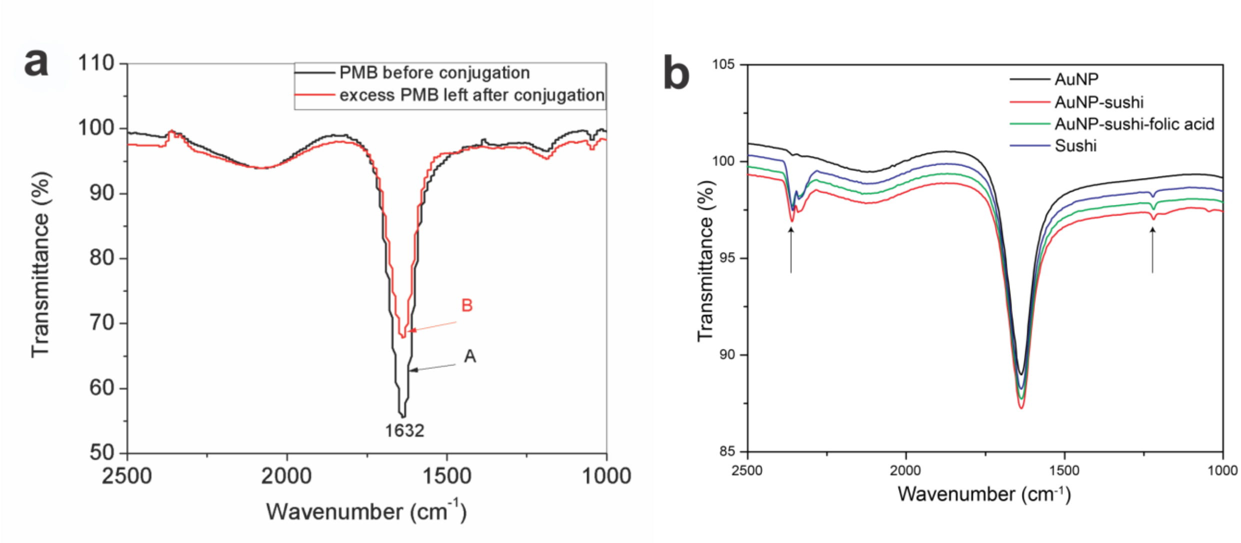


**Fig. S1. Molecular binding confirmation for PMB and sushi to AuNPs using Fourier-transform infrared (FTIR) spectroscopy: (a)** For PMB, experiments were performed on the suspension's supernatant before (A) and after (B) conjugation of PMB to the AuNPs. First, FTIR spectrum of potassium bromide (KBr) pellet was recorded in the transmission mode for background subtraction. Then, 20 µl of PMB solution in water (samples A and B) was added to the KBr pellets and kept in a vacuum oven at 70 °C for 1 h. The FTIR spectra were again recorded and the background from the blank pellet was subtracted. A drop in the peak at 1632 $cm^{-1}$ suggested successful binding of PMB to the AuNP surface. The value of 1632 $cm^{-1}$ was chosen because both the amide and carbon-carbon bonds in the aromatic benzene present in the PMB molecular structure show a characteristic absorption peak at this wavenumber. **(b)** For sushi, an attenuated total reflection (ATR) FTIR was used in which the spectra were directly recorded on sushi and AuNPs conjugated with either sushi or both sushi and folic acid. The presence of amide peaks at 1220 $cm^{-1}$ and 2360 $cm^{-1}$ in all three cases confirmed sushi conjugation to AuNPs.

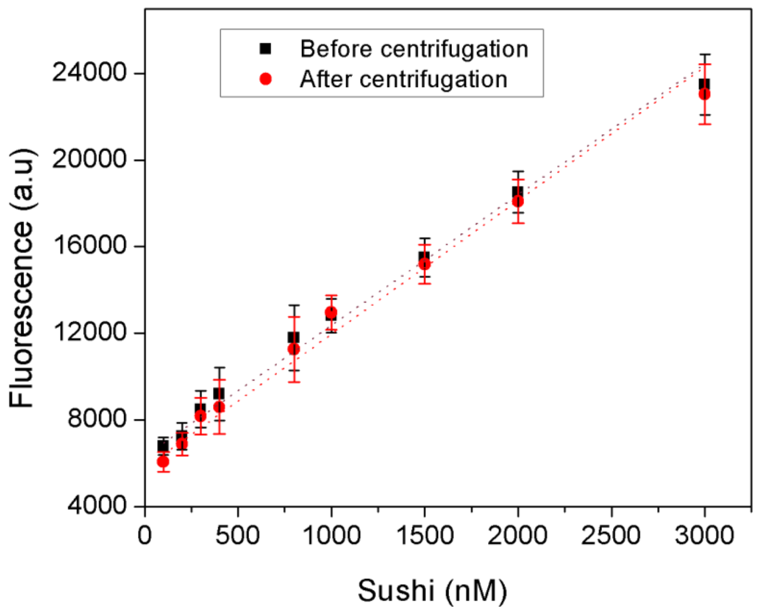


**Fig. S2. Control experiment for sushi conjugation to AuNPs:** We have shown in Fig. 2 (main text) how the OPA reagent was used to back-calculate the amount of sushi peptides conjugated to the AuNP surface. To rule out the possibility that the reduction in the fluorescence signal was not an artefact of the peptides settling by themselves during the centrifugation process, we performed a control experiment in which samples containing only peptides were taken at different dilutions. The fluorescence signal of these samples were measured in the presence of the OPA reagent by taking a small volume of the solution from the top of the eppendorf before and after centrifugation. The results in the graph above show that the calibration curves of the sushi peptides taken before (black) and after (red) centrifugation match with each other, implying that the free peptides do not settle in solution and so, the amount of conjugated peptide is established reasonably in our experiments. The lines are linear fits with least square error to the respective data.

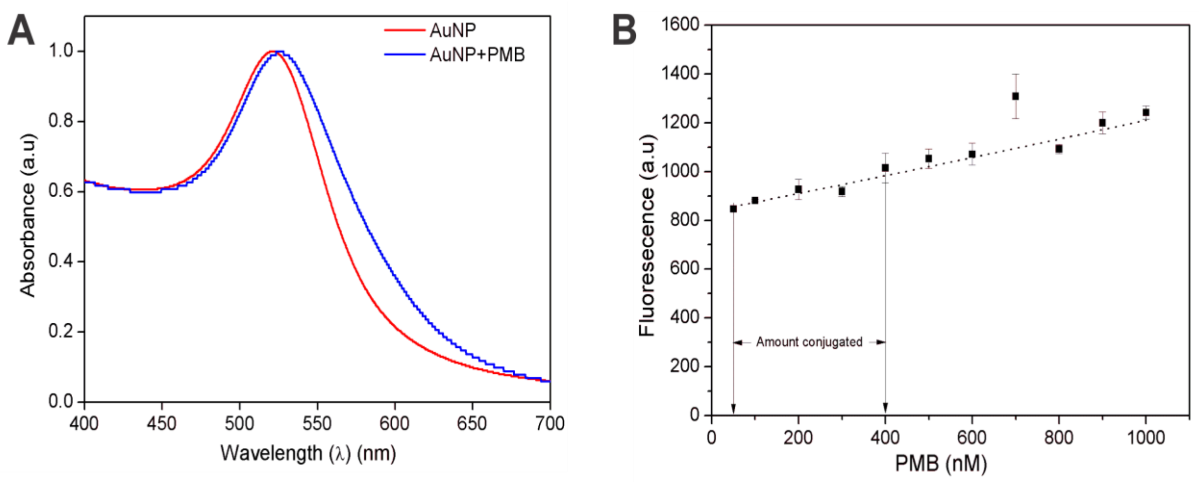


**Fig. S3. (A)** A red-shift ($\Delta\lambda_{max}$ = 5 nm) in the UV-visible absorbance spectra of the AuNPs before and after PMB addition confirmed binding. **(B)** OPA calibration chart for quantifying the amount of PMB molecules linked to AuNPs, mentioned in table 1 of main text.

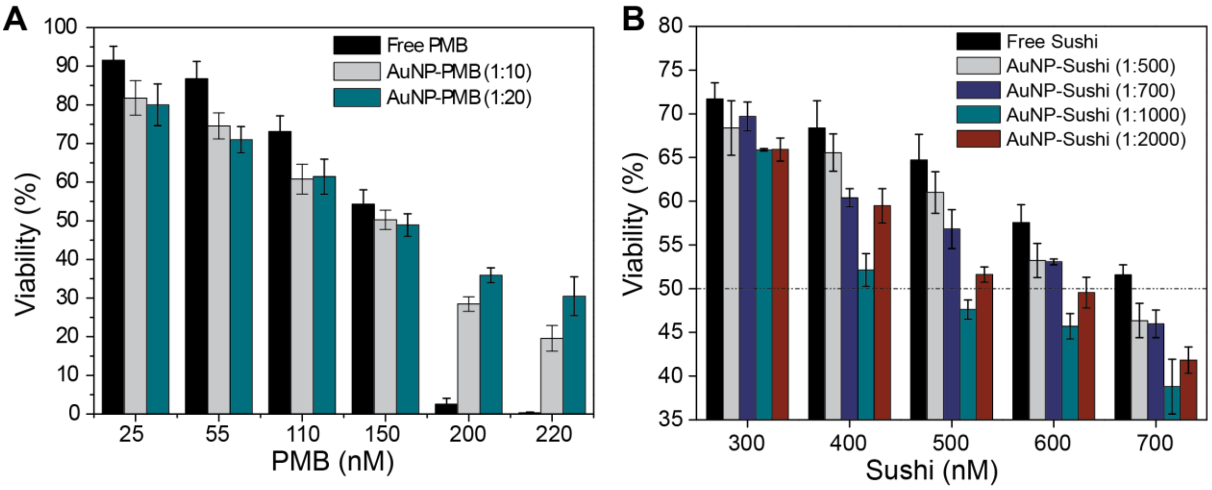


**Fig. S4. Antibacterial activity of AuNP conjugates on *E.coli*: (A)** NP-conjugated PMB was more efficacious than free drug below 150 nM but there was minimal effect of the NP:drug ratio. **(B)** NP-sushi conjugates were superior to the free drug across all concentrations especially the 1:1000 molar ratio which reduced the $IC_{50}$ value (see dotted line) by more than 25%.

The AuNP-PMB conjugates showed no major difference in the antimicrobial activity as compared to free PMB. Even doubling the amount of drug on the NPs had no significant effect on the antibacterial activity but beyond 150 nM concentration, free PMB was drastically more potent than the NPs, killing most of the cells present in the system. We believe that since PMB is already a highly potent drug; its efficacy is difficult to modulate by merely conjugating it to NPs. This is in agreement with the literature observations for a variety of other particle types and conjugation chemistries used for PMB-NP complexation[1]. In comparison, the results with sushi on *E.coli* showed a clear trend where the efficacy of the NP-bioconjugates first increased and then decreased non-monotonically as the relative molar ratio of the peptides was increased on the AuNPs. Conjugates with 1000x molar excess displayed the most favourable performance lowering the $IC_{50}$ values by as much as 28% (~ 500 nM ) when compared to the free peptide (~ 700 nM).

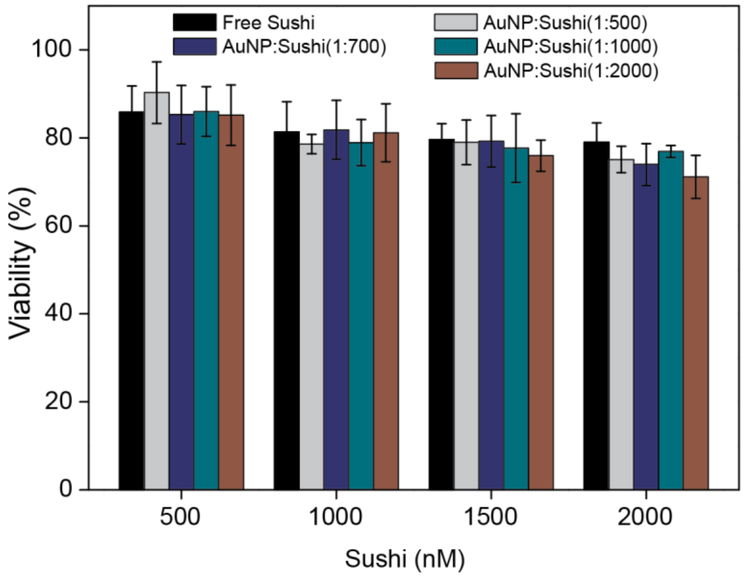


**Fig. S5.** The cytotoxicity analysis of free sushi and sushi bound to AuNPs (at different molar ratios) on HeLa cells did not show any major effect even after overnight incubation.

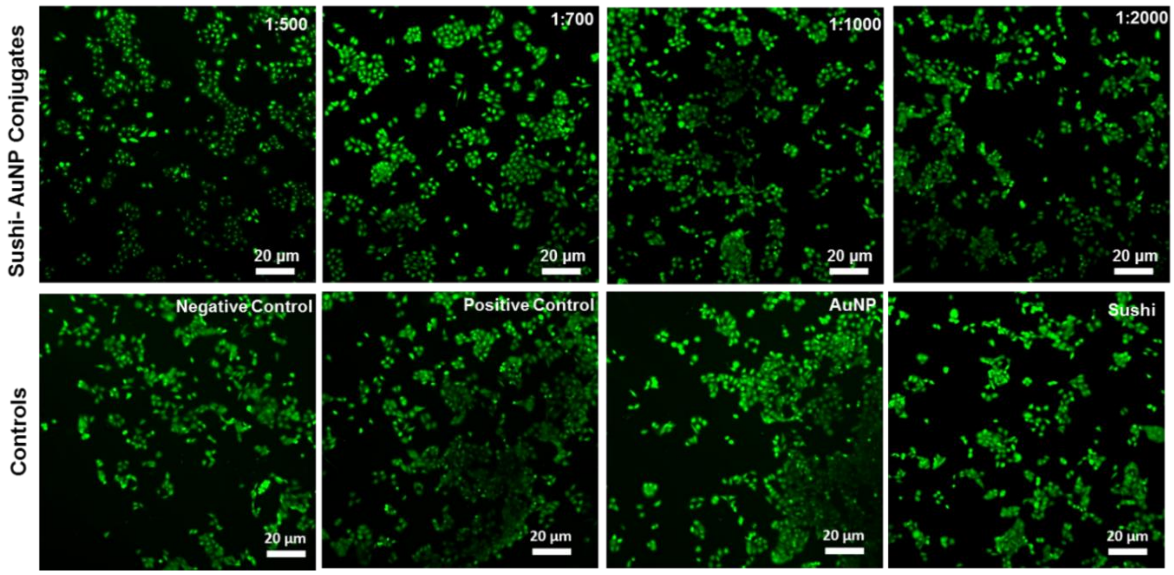


**Fig. S6.** Fluorescence microscopy images of *S. typhi*-infected HeLa cells in the presence of sushi, AuNPs (8 nM) and AuNPs conjugated with different ratios of sushi peptides. The final sushi concentration was 4 μM in all cases. The negative and positive control samples contained HeLa cells and HeLa cells infected with *S. typhi*, respectively. Images were taken with 10x objective after incubation for 5 h and staining with calcein AM. Live cells appear green in colour.

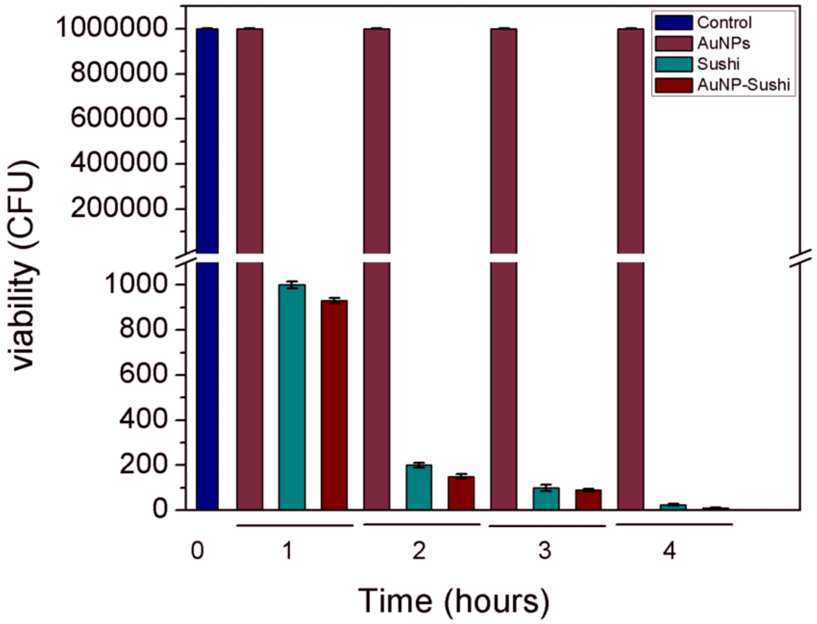


**Fig. S7. Time-bound study of antibacterial activity of sushi peptides on *E.coli* bacteria:** 2 μM peptides in the free form were applied to the bacterial suspension at 37 °C. The cells were taken out of the media every hour and plated on the agar plates which were then kept overnight in the incubator at 37 °C. The results showed a drop in cell count within the first hour and complete cell death after 4 h.

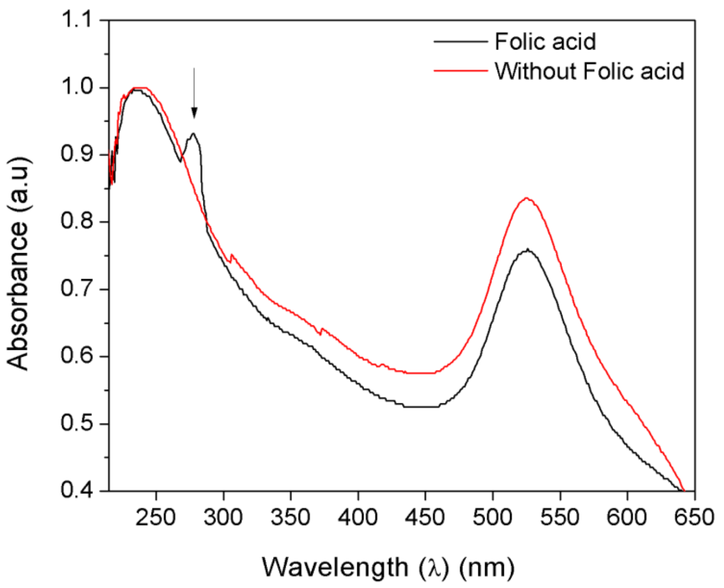


**Fig. S8.** UV-visible spectra of AuNP-sushi before and after folic acid conjugation. The appearance of a new peak at 280 nm post modification qualitatively confirmed its presence on the NPs.

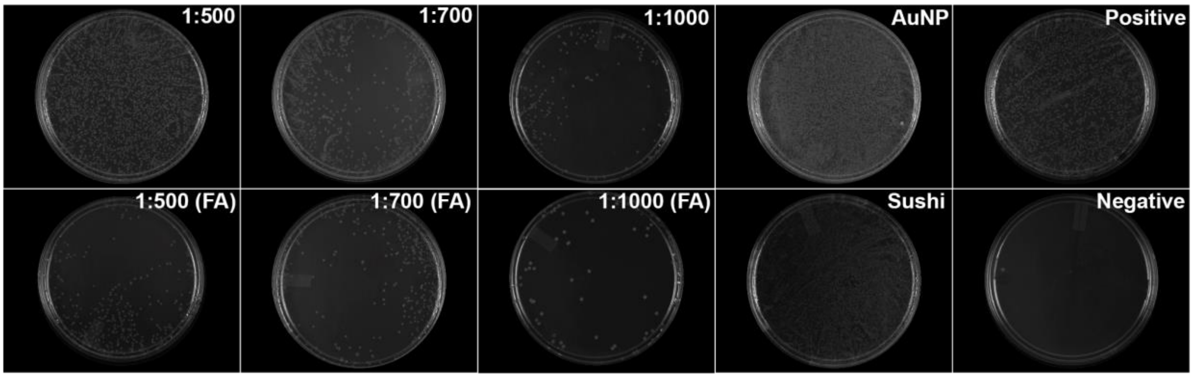


**Fig. S9. Comparison of antimicrobial activity of AuNP-sushi with and without FA-functionalization against *S.typhi*.** Actual images of plates showing colony growth after overnight incubation showing less number of colonies in corresponding FA functionalized AuNP sushi conjugates.

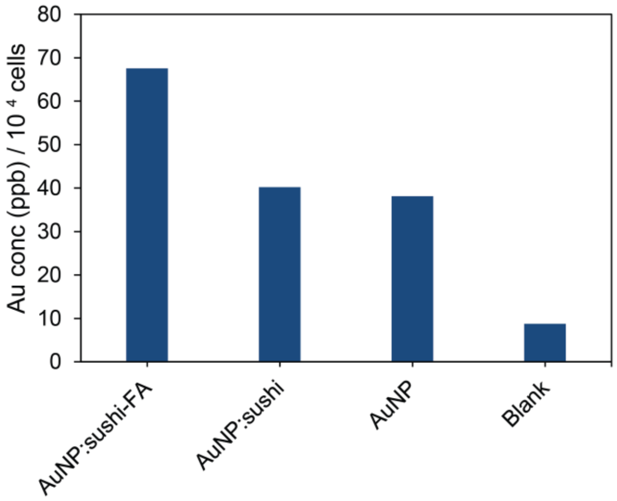


**Fig. S10.** Quantification of AuNP uptake by HeLa cells using ICP-MS, showing higher uptake of FA conjugated NPs compared to non FA conjugated NPs.

**Proposed model for AuNP-sushi and LPS interaction**

We used a geometrical model to explain our sushi peptide results. The average width of an LPS molecule on a bacterial cell membrane, *d* = 2.8 nm, was taken from the literature [2]. The mean length of a single sushi peptide molecule was estimated as 25 nm from online available tool (http://biotools.nubic.northwestern.edu/proteincalc.html) and also from manual calculations (http://www.ruppweb.org/Xray/tutorial/protein_structure.htm) based on atomic and amide bond lengths. Assuming that the peptides are distributed homogenously over the NP surface and that the packing fraction of the circles on a curved surface is the same as that on a Euclidean plane, i.e., 0.9 [3], the approximate intermolecular distance between the peptides at their base (*L*) as well as between the position of their amine groups (*L'*) was estimated for all the AuNP:sushi ratios (**Table S1**). From the calculations, it was evident that the 1:1000 ratio showed the closest commensurate match between *L'* and the per LPS molecule interphosphate distance (*d*), and hence, the most optimal interaction for achieving maximum cell death.

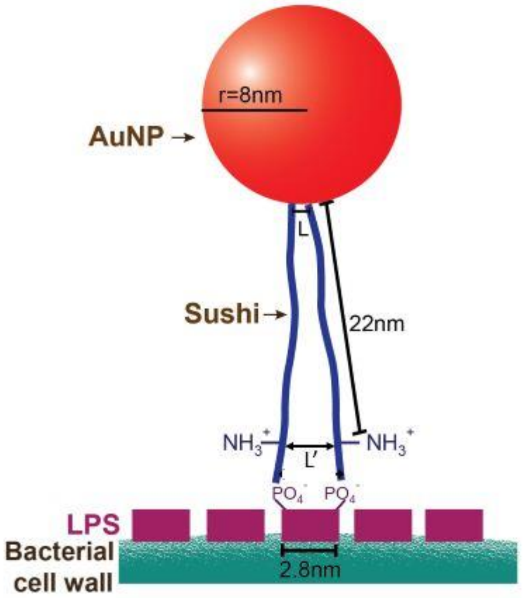


**Fig. S11.** Schematic of the proposed model

**Table S1.** Interpeptide distance between adjacent sushi molecules conjugated to a AuNP

| **AuNP:Sushi type** | **Interpeptide distance at the C-terminal, *L* (nm)** | **Interpeptide distance toward the N-terminal, *L'* (nm)** |
|---|---|---|
| 1:500 | 1.2 | 4.7 |
| 1:700 | 1 | 4 |
| 1:1000 | 0.73 | **3** |
| 1:2000 | 0.54 | 2.2 |

## References


1 Park, S., Chibli, H., Wong, J. & Nadeau, J. L. Antimicrobial activity and cellular toxicity of nanoparticle–polymyxin B conjugates. *Nanotechnology* **22**, 185101 (2011).

2 Qiao, S., Luo, Q., Zhao, Y., Zhang, X. C. & Huang, Y. Structural basis for lipopolysaccharide insertion in the bacterial outer membrane. *Nature* **511**, 108-111 (2014).

3 Chang, H.-C. & Wang, L.-C. A Simple Proof of Thue's Theorem on Circle Packing. *arXiv preprint arXiv:1009.4322* (2010).
